# Long-Term Wi-Fi Exposure From Pre-Pubertal to Adult Age on the Spermatogonia Proliferation and Protective Effects of Edible Bird’s Nest Supplementation

**DOI:** 10.3389/fphys.2022.828578

**Published:** 2022-03-11

**Authors:** Farah Hanan Fathihah Jaffar, Khairul Osman, Chua Kien Hui, Aini Farzana Zulkefli, Siti Fatimah Ibrahim

**Affiliations:** ^1^Department of Physiology, Faculty of Medicine, Universiti Kebangsaan Malaysia (UKM), Kuala Lumpur, Malaysia; ^2^Faculty of Health Sciences, Universiti Kebangsaan Malaysia (UKM), Kuala Lumpur, Malaysia

**Keywords:** children, gonadotropin, mitosis, spermatogonia, sperm, Wi-Fi

## Abstract

Children are vulnerable to the radiofrequency radiation (RFR) emitted by Wi-Fi devices. Nevertheless, the severity of the Wi-Fi effect on their reproductive development has been sparsely available. Therefore, this study was conducted to evaluate the Wi-Fi exposure on spermatogonia proliferation in the testis. This study also incorporated an approach to attenuate the effect of Wi-Fi by giving concurrent edible bird’s nest (EBN) supplementation. It was predicted that Wi-Fi exposure reduces spermatogonia proliferation while EBN supplementation protects against it. A total of 30 (*N* = 30) 3-week-old Sprague Dawley weanlings were divided equally into five groups; Control, Control EBN, Wi-Fi, Sham Wi-Fi, and Wi-Fi + EBN. 2.45 GHz Wi-Fi exposure and 250 mg/kg EBN supplementation were conducted for 14 weeks. Findings showed that the Wi-Fi group had decreased in spermatogonia mitosis status. However, the mRNA and protein expression of c-Kit-SCF showed no significant decrease. Instead, the reproductive hormone showed a reduction in FSH and LH serum levels. Of these, LH serum level was decreased significantly in the Wi-Fi group. Otherwise, supplementing the Wi-Fi + EBN group with 250 mg/kg EBN resulted in a significant increase in spermatogonia mitotic status. Even though EBN supplementation improved c-Kit-SCF mRNA and protein expression, the effects were insignificant. The improvement of spermatogonia mitosis appeared to be associated with a significant increase in blood FSH levels following EBN supplementation. In conclusion, the long-term Wi-Fi exposure from pre-pubertal to adult age reduces spermatogonia proliferation in the testis. On the other hand, EBN supplementation protects spermatogonia proliferation against Wi-Fi exposure.

## Introduction

Amid the pandemic COVID-19 outbreak, Wi-Fi has become a daily necessity rather than a privilege ([MCMC] Malaysian Communications and Multimedia Commission, 2020). The worldwide lockdown had caused an increment of 500 and 155% of Wi-Fi users among children in the United States and Malaysia, respectively ([MCMC] Malaysian Communications and Multimedia Commission, 2020). In Malaysia, this involves children aged 5–17 years old ([MCMC] Malaysian Communications and Multimedia Commission, 2020). Children nowadays have been using Wi-Fi since before puberty and may continue to do so until adulthood. Therefore, the trend of using Wi-Fi early in childhood causes the children to receive radiofrequency radiation (RFR) emitted by the Wi-Fi devices for a more extended period than it will be to adults (Stewart et al., 2000; Moon, 2020).

In view of the fact that the testis is a sensitive organ to the RFR emitted by Wi-Fi (Zhu et al., 2014), the focus of this study was to evaluate the effect of Wi-Fi exposure on the testis when it receives early Wi-Fi exposure since childhood. The testis is an essential organ for male gamete production through spermatogenesis (Nishimura and L’Hernault, 2017). Spermatogenesis is a continuous process that begins at the onset of puberty to produce millions of spermatozoa (Feng et al., 2014). This process involves strict regulation of multifactorial niche in the testis microenvironment (Hai et al., 2014).

Before the onset of the first meiosis during puberty, spermatogonial stem cells (SSCs) located in the seminiferous tubule of the testis should undergo two processes. The first is mitosis for the maintenance of the SSC pool, and the second is differentiation to produce differentiated spermatogonia progenitor (Kanatsu-Shinohara and Shinohar, 2013). The differentiated spermatogonia are marked by the expression of c-Kit, a type III tyrosine kinase receptor (Ohta et al., 2000; Zhang et al., 2011; Babaei et al., 2016). c-Kit is a 145 kDa transmembrane glycoprotein coded by a gene located at the dominant white spotting (W) locus on chromosome 4q11-q12 in humans (Badr et al., 2018) and chromosome 5 in mice (Gomes et al., 2018).

The ligand for c-Kit is known as stem cell factor (SCF), a 45 kDa glycoprotein secreted by Sertoli cells and encoded by Steel (Sl) locus on chromosome 12 in humans and chromosome 10 in mice (Wiesner et al., 2008; Mansuroglu et al., 2009). Interaction and communication of c-Kit-SCF are crucial for the survival and proliferation of the differentiated spermatogonia and the onset of spermatogenesis (Prabhu et al., 2006). Subsequently, the proliferation of the differentiated spermatogonia is a critical stage to dictate the number of spermatogonia that will enter meiosis and determine the number of sperm yields (Xu et al., 2016).

Besides c-Kit-SCF interaction, important reproductive hormones also influence spermatogonia proliferation (Khanehzad et al., 2021). As the Wi-Fi exposure was done from pre-pubertal to adult age, the probability of hormonal disturbance on the testosterone, follicle-stimulating hormone (FSH), and luteinizing hormone (LH) is quite likely. However, whether early Wi-Fi exposure could impair spermatogonia proliferation and these prominent factors regulating them is still unknown.

Besides evaluating the effect of pre-pubertal Wi-Fi exposure on the testis, this study also incorporated edible bird’s nest (EBN) supplementation to overcome the expected negative impact of Wi-Fi exposure. EBN is a bird’s nest made of male swiftlet saliva, *Aerodramus fuciphagus*, particularly during the breeding season (Kuntjoro and Rachmadiarti, 2020). It is composed mainly of protein, carbohydrates, and a small amount of fat (Chua and Zukefli, 2016). Our previous study demonstrated a dose-dependent increase of sperm concentration, sperm motility, as well as testosterone, FSH, and LH serum hormonal level upon EBN supplementation (Jaffar et al., 2021).

There are two reasons why this study incorporates EBN. Firstly, EBN was reported to have a positive proliferative effect in *in vitro* study (Aswir and Wan Nazaimoon, 2011; Abidin et al., 2011; Roh et al., 2012). Second, EBN was also reported containing a few male reproductive hormones such as FSH, LH, and testosterone (Ma and Liu, 2012a) and showed potential as an alternative treatment for erectile dysfunction (Ma et al., 2012). These EBN characteristics align with the current study objective and therefore becomes the reason for this selection interest.

Hereby, this study was done to evaluate the effect of long-term Wi-Fi exposure from pre-pubertal until adult age and its impact on spermatogonia proliferation, several spermatogonia proliferation regulatory factors, and the protective effect of EBN supplementation against the Wi-Fi exposure. This study is imperative in understanding the consequences of long-term Wi-Fi exposure on children’s reproductive development and the approach to overcome the Wi-Fi effect with continuous Wi-Fi exposure.

## Materials and Methods

### Animals

Thirty (*N* = 30) male Sprague–Dawley rats at the age of 3 weeks with an initial body weight of 45 ± 5 g were acquired from the Laboratory Animal Research Unit (LARU) of the National University of Malaysia (UKM). This 3-week-old male rat pups representing 7-year-old children. Wi-Fi exposure was conducted for 14 weeks until the pups reached 17-week-old. The 17-week-old rats represent 33-year-old humans who have matured reproductive systems. This age comparison was performed according to the pre-pubertal period of rats compared to humans (Sengupta, 2013).

The rats were divided randomly and equally into five groups (Table 1), with six rats (*n* = 6) in each group. Each rat was housed individually in a plastic cage (43 cm length × 16 cm wide × 29 cm height) and have the privilege to move freely inside the cage without any movement restriction. The rats were fed with respective food pellets, supplied with clean water *ad libitum*, and kept in 12 h light: dark cycle at the ambient temperature of 22°C ± 5°C. In this study, all animal procedures were approved by the National University of Malaysia (UKM). The approval reference number for this study was FISIO/PP/2018/SITI FATIMAH/28-MAR./908-MAR.-2018-DEC.-2020.

**TABLE 1:** Description of experimental group.

Group	Description
Control group	No Wi-Fi exposure from a Wi-Fi router + normal food pellet
Control EBN	No Wi-Fi exposure from a Wi-Fi router + EBN enriched food pellet
Wi-Fi	Wi-Fi exposure from an active Wi-Fi router + normal food pellet
Sham Wi-Fi	Exposure from an inactive Wi-Fi router + normal food pellet
Wi-Fi + EBN	Wi-Fi exposure from an active Wi-Fi router + EBN enriched food pellet

### Wi-Fi Exposure Setting

A Wi-Fi router, namely, TP-LINK AC750 Wireless Dual Band Wi-Fi Router Archer C20 (Shenzhen, China), was used. This Wi-Fi router consists of three omnidirectional antennas. Two antennas generate 2.45 GHz Wi-Fi frequency, while one antenna supports dual frequencies, 2.45 and 5 GHz. Only 2.45 GHz Wi-Fi frequency operating at 802.11b/g/n standards was used.

**FIGURE 1:**
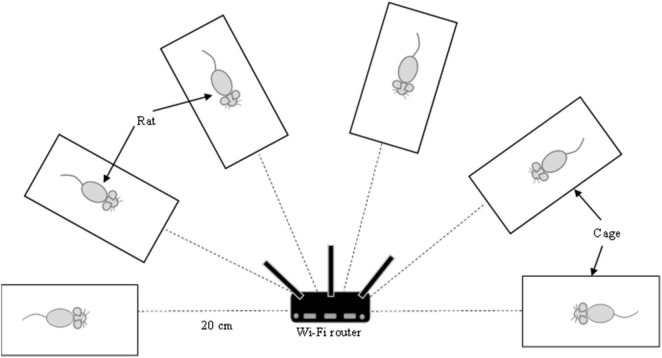
The experimental setting for Wi-Fi exposure. Wi-Fi router was placed at 20 cm distance from the animal cages. The animal was caged individually without any movement restriction. Exposure was conducted 24 h daily for 14 consecutive weeks.

The Wi-Fi router was placed at a 20 cm distance from the animal cages (Figure 1). Wi-Fi group was exposed to a router maintained in an active state by constant communication (ping protocol via Bitvise SSH client software v8.18) with a Raspberry Pi computer. Ten pings were sent per minute throughout the exposure. In the Sham Wi-Fi group, the Wi-Fi router was maintained without the ping and considered to be in an inactive state. Exposure was conducted 24 h daily for 14 consecutive weeks.

Based on the Maximum Permissible Exposure report of TP-LINK AC750 Wireless Dual Band Wi-Fi Router Archer C20, the specific absorption rate (SAR) is approximately 0.41 W/kg.

### Edible Bird’s Nest Supplementation

The raw EBN was obtained from an identified swiftlet’s house at Bera, State of Pahang, Peninsular Malaysia. The collected EBN were then processed, freeze-dried, and provided by Glycofood Sdn Bhd, Malaysia.

EBN 250 mg/kg supplementation was given to the Control EBN and Wi-Fi + EBN groups. The dose chosen and the method of supplementation was done based on our previous report (Jaffar et al., 2021).

### Animal Euthanization and Tissue Sampling

After 14 weeks of Wi-Fi exposure, each animal was euthanized with a Ketamine-Tiletamine-Xylazine (KTX) cocktail intraperitoneally. Blood was drawn immediately from retro-orbital sinus and collected in BD Vacutainer SST II Advance Plus Blood Collection Tube (BD, United States). The blood was left undisturbed for at least 30 min at room temperature, and the collection tubes were centrifuged at 1,500 g for 10 min at 4°C. The obtained serum was aliquoted and stored at −80°C until analysis.

Both sides of the testis, epididymis and seminal vesicle were carefully dissected and cleaned from surrounding adipose tissue before weighing. The organ coefficient of each dissected reproductive organ was expressed according to the equation: the wet weight of organ (g)/body weight (g) × 100 (Feng et al., 2015). The right testis was fixed in 10% buffered neutral formalin (Merck, Germany), processed with standard protocol, and embedded in paraffin wax (Merck, Germany). The left testis was immediately snap-frozen and stored at −80°C until analysis.

### Evaluation of Spermatogonia Mitosis Status by Direct Immunofluorescence

Serial sections of 3-μm thick paraffin-embedded testis were prepared and mounted on poly-L-lysine slides. The sections were deparaffinized in xylene and then rehydrated in graded alcohols.

The tissue sections were heated in a microwave oven in pre-heated 10 mM sodium citrate buffer pH 6.0 for 5 min for antigen retrieval and permeabilized with 0.1% Triton-X 100 in PBS for 30 min. The slides were washed three times with PBS before incubation in Blocking One Solution (Nacalai Tesque, Kyoto, Japan) for 10 min to block non-specific binding. The slides were rewashed with PBS three times. The tissue sections were incubated overnight at 4°C with mouse monoclonal phosphohistone 3 (pHH3) antibody conjugated with Alexa Flour 594 (Santa Cruz Biotechnology, Texas, United States) at a dilution of 1:100.

After antibody binding, the slides were washed three times with PBS. DNA was counterstained with 10 μg/mL Hoechst 33342 and observed using Nikon Eclipse Ni fluorescent microscope under 400 × magnification. About 10–15 random fields were captured using Nikon Y-T TV. The intensity of the staining of 20 random seminiferous tubules was measured using Image J software v1.52a. The steps were repeated for rectum adenocarcinoma tissue (Kim et al., 2018) as a positive control for the staining.

### qPCR Analysis of c-Kit and Stem Cell Factor in the Testis

Total RNA was extracted from the left testis using the NucleoSpin^®^RNA kit (Macherey-Nagel, Düren, Germany). The RNA was eluted with 60 μL of RNase-free water, and its concentration was determined using a NanoDrop DS-11 + Spectrophotometer (DeNovix, Wilmington, United States). Total RNA was converted into a more stable single-strand cDNA using commercially High-Capacity cDNA Reverse Transcription Kits (Applied Biosystems, California, United States). RNA stabilization was done by mixing 1,000 ng of total RNA in a master mix containing RT buffer, 100 mM dNTP mix, RT random primers, and MultiScribe^®^ Reverse Transcriptase. The reaction was conducted for 10 min at 25°C, 120 min at 37°C, and 5 min at 85°C.

Quantitative Real-Time PCR (qPCR) reactions were conducted using the PrecisionPLUS qPCR master mix (Primer Design, United Kingdom). Dilution of 1:5 and 1:50 cDNA was applied for c-Kit and SCF, respectively. Approximately 1 μL of the respective cDNA dilution and 1 μL of each primer (Table 2) were added into the master mix. A final reaction volume of 20 μL was run in CFX96TM Real-Time System attached to C1000 Thermal Cycler (Bio-Rad, United States) under the following conditions: 95°C for 2 min, followed by 40 cycles of 95°C for 10 s, and 60°C for 60 s. A final melting step was performed under the following conditions: 65–95°C for 5 s, with 0.5°C increments, to ensure primer dimers’ absence and confirm reaction specificity.

**TABLE 2:** Sequence and code for primers of targeted and housekeeping genes.

Gene	Primer sequence 5′–3′/Primer code	NCBI accession number	Expected product size (bp)
c-Kit	QT00194145	NM_022264, XM_006250909	103
SCF	QT00411285	NM_021843, NM_021844, XM_008765339, XM_008765340	129
β-actin	Forward: TCT GTG TGG ATT GGT GGC TCT A	NM_031144_3	69
	Reverse: CTG CTT GCT GAT CCA CAT CTG		

All reactions were run in triplicate. Each sample’s threshold cycle value (Ct) was measured from the fluorescence signal at the end of every extension cycle. β-Actin was used as a housekeeping gene. The Ct values were used to calculate the relative expression levels compared with the housekeeping gene expression.

Standard plots were constructed for each target gene by using specific primers (Table 2). Each standard curve was generated by linear regression of the plotted points. The standard curves exhibited correlation coefficients not less than 0.99, and the efficiencies ranged between 100 and 110%.

### Evaluation of c-Kit and Stem Cell Factor Protein Expression by Western Blot

The frozen rat testis was thawed and homogenized in radioimmunoprecipitation assay buffer (RIPA) buffer containing protease inhibitors. The samples were centrifuged at 12,000 rpm for 10 min at 4°C. The supernatant was extracted, aliquoted, and stored at 80°C. Bradford assay was performed to determine protein concentration in each homogenate sample. About 0.2 g of the protein sample was mixed with SDS 5 × loading buffer (Elabscience, Wuhan, China), incubated at 70°C for 5 min, and centrifuged at 12,000 rpm for 30 s. The proteins were electrophoresed on 10% polyacrylamide gels for c-Kit and 8% polyacrylamide gels for SCF.

The membranes were blocked using 5% (w/v) skim milk in Tween-containing Tris-buffered saline [TBST; 145.4 mM NaCl, 10 mM Tris-base, 0.1% (v/v) Tween20, pH 7.5] for 1 h. Then it was incubated in rabbit c-Kit polyclonal (1:1,000 in 5% skim milk) (Elabscience, Wuhan, China) and rabbit SCF polyclonal (1:1,000 in 5% skim milk) (Elabscience, Wuhan, China) primary antibodies overnight at 4°C. The membrane was washed with TBST and incubated with goat anti-rabbit Ig-G (H + L) conjugated with horseradish peroxidase (HRP) (1:5,000 in 2% skim milk) (Elabscience, Wuhan, China) for 1 h at room temperature. The membrane was rewashed with TBST, and the immunoreactive bands were detected using the Excellent Chemiluminescent Substrate (ECL) Kit (Elabscience, Wuhan, China). The membranes were stripped and re-blotted with an anti-β-actin antibody to determine equal loading. Protein detection was then done on the membrane. It was performed using Gel Doc Amersham Imager 600 (GE Healthcare, United Kingdom). Digital images from the latter imager were immediately analyzed using Image J v. 1.52a. The band intensities of the protein were normalized to that of β-actin.

### Serum Testosterone, Follicle-Stimulating Hormone, and Luteinizing Hormone Determination by ELISA

Serum testosterone was measured by competitive enzyme-linked immunosorbent assay (ELISA, Elabscience, Wuhan, China). Serum FSH and LH were measured by sandwich ELISA (Elabscience, Wuhan, China). Intra-assay and inter-assay variability (CV) for all ELISA kits was less than 10%.

All the assay procedures were conducted in duplicate according to the kit’s instructions. In brief, 50 μL of the standard and serum were pipetted into a 96 well testosterone ELISA plate. The well was immediately added with 50 μL of biotinylated antibody, sealed, and incubated for 45 min at 37°C.

For FSH and LH hormone analyses, 100 μL of the standard and serum were pipetted into a 96 well ELISA plate. The plate was sealed and incubated for 90 min at 37°C, and all liquid was removed before adding 100 μL of respective biotinylated antibodies into the well. The well was then incubated for 1 h at 37°C.

After incubation with the respective biotinylated antibody, all the solution was removed from the well and washed three times. About 100 μL of horseradish peroxidase (HRP)-conjugated avidin was added into each well. The well was further incubated for another 30 min at 37°C and washed five times. The colorimetric reaction was allowed by adding 90 μL of the substrate reagent, covered from light, and incubated for another 15 min. The reaction was terminated by adding 50 μL of the stop solution. Absorbance was recorded at 450 nm. Testosterone, FSH, and LH serum levels were interpolated from an eight-point standard curve plotted according to a four-parameter logistic (4PL) by using MyAssays.com.

### Statistical Analysis

Statistical analysis was performed using SPSS version 22.0 software (SPSS Inc., Chicago, IL, United States). One-way analysis of variance (ANOVA) followed by Tukey’s *post hoc* analysis was conducted due to the data’s normal distribution and variance homogeneity. The Kruskal-Wallis H test evaluated immunofluorescence intensity and sperm chromatin integrity because of the lack of normality of the data distribution. A *p*-value < 0.05 was considered statistically significant.

## Results

### Organ Coefficient

No significant differences in the organ coefficients of the testis, epididymis, and seminal vesicles were found among the groups (Table 3).

**TABLE 3:** Organ coefficient of testis, epididymis and seminal vesicle in each group.

Group/Organ	Testis	Epididymis	Seminal vesicle
Control	0.40 ± 0.02	0.17 ± 0.00	0.43 ± 0.05
Control EBN	0.34 ± 0.02^a^	0.16 ± 0.01	0.39 ± 0.02
Wi-Fi	0.38 ± 0.01	0.16 ± 0.01	0.36 ± 0.04
Sham Wi-Fi	0.36 ± 0.01	0.15 ± 0.00	0.39 ± 0.02
Wi-Fi + EBN	0.32 ± 0.01^a^,^b^	0.14 ± 0.00^a^	0.39 ± 0.02

*Organ coefficient refers to the wet weight of organ (g)/body weight (g) × 100. Data is presented as mean ± SEM (n = 6).*

*^a^Represents significant difference compared to the Control group.*

*^b^Represents significant difference compared to the Wi-Fi group.*

### Spermatogonia Mitosis Status

**FIGURE 2:**
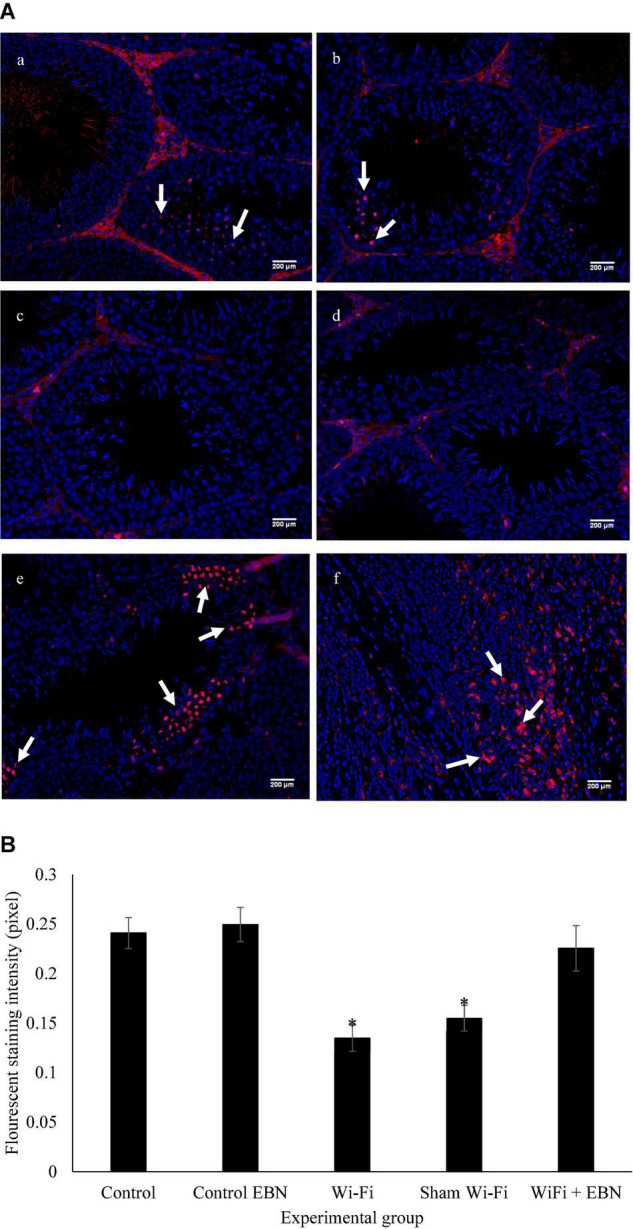
**(A)** Immunofluorescence staining on sections of the testis for the expression of pHH3 by using Alexa Flour 594, with excitation and emission wavelengths of 590 and 618 nm, respectively. Positive staining is represented by red fluorescence signal (arrow). **(a)** Control group; **(b)** Control EBN group; **(c)** Wi-Fi group; **(d)** Sham Wi-Fi group; **(e)** Wi-Fi + EBN group **(f)** staining positive control by using rectum adenocarcinoma tissue section. For each section, Hoechst 33342 was used to counterstain the cell nuclei, as indicated by the blue signal. All observations were conducted under 200× magnification. **(B)** Immunofluorescence intensity measured by Image J is presented as median ± SEM (*n* = 6). Kruskal-Wallis H test showed significant differences * among the groups (Chi-square = 24.84, *p* = 0.000, df = 2).

The mitosis status of spermatogonia was evaluated by pHH3 expression, represented by red immunofluorescence staining (Figure 2A). The immunofluorescence staining of the testis tissue in each group demonstrated that Wi-Fi and Sham’s groups exhibited reduced immunofluorescence red signal. The intensity of the red immunofluorescence signal was measured by Image J software and evaluated by the Kruskal-Wallis H test. A significant difference in the signal intensity was found among the groups (Chi-square = 24.84, *p* = 0.000, df = 2) (Figure 2B).

### Evaluation of c-Kit and Stem Cell Factor mRNA Expression by qPCR

**FIGURE 3:**
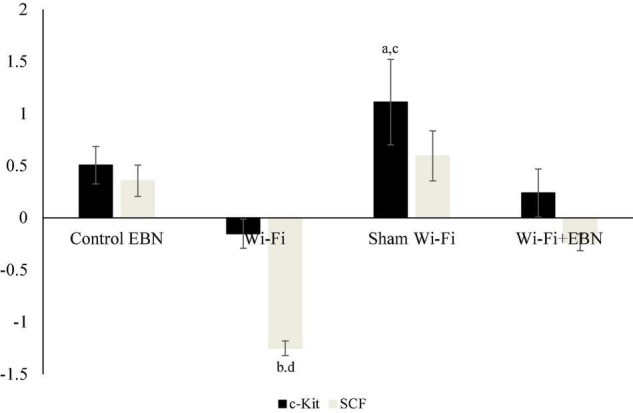
Log_2_ fold change of c-Kit and SCF mRNA expression in each group. mRNA expression was determined by qPCR after normalization with β-actin housekeeping gene. Data are expressed as fold change variation relative to the Control group (0 baselines) and presented as mean ± SEM (*n* = 6). ^a^Represents a significant difference compared with the Control group. ^b^Represents a significant difference compared with the Control EBN group. ^c^Represents a significant difference compared with the Wi-Fi group. ^d^Represents a significant difference compared with the Sham Wi-Fi group.

There were no significant changes in c-Kit and SCF mRNA expression in the Wi-Fi group compared with the Control group. However, the mRNA expression levels of c-Kit (*p* = 0.007) and SCF (*p* = 0.000) in the Wi-Fi group showed a statistically significant difference compared with those in the Sham group. The c-Kit (*p* = 0.015) mRNA expression was significantly higher in the Sham group than in the Control group (Figure 3). The SCF mRNA expression in the Sham group showed a similar trend with c-Kit mRNA expression, but it was not statistically significant compared with the Control group.

### Evaluation of c-Kit and Stem Cell Factor Protein Expression by Western Blot

**FIGURE 4:**
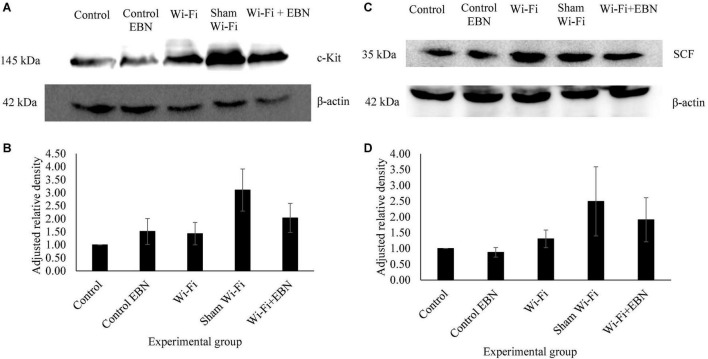
Protein expression of c-Kit and SCF in each experimental group. **(A,C)** Are the representative blots of five different rats. The β-actin lanes belong to the same blot and have the same film exposure. **(B,D)** Represents relative intensities compared with β-actin measured by ImageJ. Data are expressed as mean ± SEM (*p* > 0.05; One-way ANOVA, *n* = 5).

We further examined the protein levels of c-Kit (Figures 4A,B) and SCF (Figures 4C,D) in each group. The results of relative intensities with β-actin as standard showed that the protein expression levels of c-Kit (*p* = 0.977) and SCF (*p* = 0.996) in the Wi-Fi group showed no significant changes compared with those in the Control group. The c-Kit and SCF protein expression levels increased in the Sham group but were not statistically significant compared with those in Control (c-Kit *p* = 0.071; SCF *p* = 0.428) and Wi-Fi group (c-Kit *p* = 0.205; SCF *p* = 0.634). These patterns of protein expression indicate that changes in protein and mRNA levels are somehow parallel.

### Serum Testosterone, Follicle-Stimulating Hormone, and Luteinizing Hormone

**FIGURE 5:**
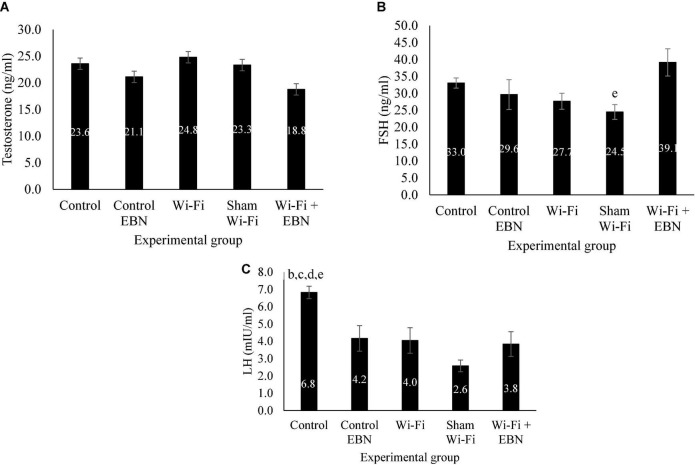
Serum hormonal level of male reproductive hormones in each group following exposure. Data are presented as mean ± SEM (*n* = 6). **(A)** Serum testosterone level in each group following the Wi-Fi exposure. No significant difference was noted among the groups. **(B)** Serum FSH level in each group following the Wi-Fi exposure. ^e^Significant difference compared with Wi-Fi + EBN group. **(C)** Serum LH level in each group following the Wi-Fi exposure. ^b^Significant difference compared with the Control EBN group (*p* = 0.030), ^c^Significant difference compared with the Wi-Fi group (*p* = 0.039), ^d^significant difference compared with the Sham Wi-Fi group (*p* = 0.000), and ^e^significant difference compared with the Wi-Fi + EBN group (*p* = 0.011).

Serum testosterone level was not statistically different among the groups (Figure 5A). There was a gradual decrease in the serum FSH level but was not significant among the groups (Figure 5B). The serum LH level significantly decreased in Wi-Fi (*p* = 0.039) and Sham (*p* = 0.000) groups compared with that in the Control group (Figure 5C).

## Discussion

The discussion was separated into two sections to make it easier to understand. The first section will be explaining the effect of Wi-Fi exposure on the male reproductive system of Sprague Dawley pups. At the same time, the second section will demonstrate the possibility of EBN to overcome the effect of Wi-Fi exposure.

### The Effect of Wi-Fi Exposure on Male Children Reproductive System

To date, only two studies have reported the effect of Wi-Fi exposure on the testis of rat pups, which represent children (Özorak et al., 2013; Šimaiová et al., 2019). Both studies only evaluated the effect of Wi-Fi up to several weeks of life. Nevertheless, the use of Wi-Fi is more protracted in the current pattern and often in a consistent manner that can seem to take up eternity. In this study, we applied long-term Wi-Fi exposure from pre-pubertal until adulthood. Findings showed that all the animals successfully achieved puberty following 2 weeks of Wi-Fi exposure. This is indicated by the enlargement of the testis. Long-term Wi-Fi exposure also did not affect the testis, epididymis, and seminal vesicle coefficient, which was not significantly different among the groups. However, both Wi-Fi and Sham Wi-Fi groups had caused a substantial decrease in the spermatogonia mitosis status in the testis. As mitosis of the spermatogonia is an essential step that contributes to the sperm concentration yields, this finding reflected the consistent report on sperm concentration reduction following Wi-Fi exposure in previous studies (Mahmoudi et al., 2015; Saygin et al., 2016).

This study evaluated the possible regulatory factors that may cause the reduction in the spermatogonia mitosis status following Wi-Fi exposure. Evaluation of the c-Kit-SCF regulatory factor showed a non-significant decrease in both mRNA expression in the Wi-Fi group compared to the Control group. On the other hand, it is interesting to note a significant increase of c-Kit-SCF mRNA expression in the Sham Wi-Fi group compared to the Control group. The mRNA expression of c-Kit-SCF was further evaluated by their respective protein expression. A slight reduction in c-Kit and SCF mRNA expression in the Wi-Fi group did not affect the protein expression, and it is comparable to the expression in the Control group. On the other hand, the substantial increase in c-Kit and SCF mRNA expression in the Sham Wi-Fi group indicated an increase in the levels of the respective proteins. However, the increased expression of an individual protein in the Sham Wi-Fi group was also insignificant compared to the Control group.

The changes in the c-Kit-SCF mRNA and protein expression in both Wi-Fi and Sham Wi-Fi groups might relate to the different settings applied for the exposure. Wi-Fi group received Wi-Fi signals from an active router (data transfer is on) while Sham Wi-Fi group was only exposed to inactive Wi-Fi router (data transfer is off). It seems that the active Wi-Fi router had caused a higher RFR compared to the passive Wi-Fi router. This postulation was consistent with a previous study reported by Pachón-García et al. (2015) where they found that the maximum background exposure increases when the Wi-Fi network is in operation compared to when the Wi-Fi network is off.

The greater RFR emitted by an active router may cause the decrease of c-Kit-SCF mRNA expression. On the other hand, the probability of having a lower RFR emitted from an inactive Wi-Fi router may promote a positive effect in the testis and cause the increase of c-Kit-SCF mRNA expression in the Sham Wi-Fi group. The postulation about these findings seems to be consistent with biphasic dose-response explained in radiation hormesis theory (Jin et al., 2020). Although this theory is applied to ionizing radiation, it also suggests that low doses of radiation may exert protective effects and induce beneficial outcomes. In contrast, higher doses lead to detrimental effects (Jargin, 2020).

Delavarifar et al. (2020) observed a similar discovery of beneficial low-dose radiation generated by Wi-Fi routers. Their study applied a shorter exposure time (2 h daily for 4 days) at a greater distance from the router (100 and 150 cm). This setting resulted in a lower specific absorption rate (SAR). These exposure settings increased the sperm concentration and sperm histomorphometric parameters in Busulfan-induced oligospermic mice (Delavarifar et al., 2020).

To the best of our knowledge, this study is the first to report the association of Wi-Fi exposure with the c-Kit-SCF regulation system in the testis. Although the finding seems to be in line with radiation hormesis theory, we cannot rule out the possibility of the detrimental effect when c-Kit and SCF are overexpressed (Cardoso et al., 2017). We do not have the privilege to discuss our results further due to a lack of supporting information on bio-positive and/or bio-negative effects observed. Further studies may shed light on the exact mechanisms of how low and high RFR emitted by the Wi-Fi router affects the testis.

Since the protein expression of c-Kit and SCF in both groups was not affected, the communication between c-Kit and SCF has still occurred despite the Wi-Fi exposure given. However, the proliferation of the spermatogonia was significantly reduced in both groups. It thus suggested that the c-Kit-SCF crosstalk may have failed. As there were no changes in both proteins’ expression, the reduction in spermatogonia mitosis may have been related to the protein structural changes upon Wi-Fi exposure. The structural changes of a protein due to EMF exposure have been previously proven by Todorova et al. (2016) Thus, the protein structural changes may have caused the impairment in the crosstalk between c-Kit-SCF and subsequently affected the spermatogonia mitosis status.

As the regulation of the spermatogonia proliferation involved a very complex interaction with other biomolecules, there is a possibility that factors other than c-Kit-SCF may also have been affected by the Wi-Fi exposure. Given that Wi-Fi exposure was conducted before puberty, major male reproductive hormones may have been affected following the exposure. Findings showed a decrease in gonadotropin, FSH and LH serum levels in Wi-Fi and Sham Wi-Fi groups. Whether serum FSH and LH levels are affected by the distinctive exposure of Wi-Fi, or a sign of a typical negative feedback mechanism remains unclear.

However, in terms of FSH action, it is the primary hormone that regulates germ cell numbers by increasing the proliferation of spermatogonia (Ding et al., 2011). So, a decrease in spermatogonia mitosis status may have enhanced the FSH secretion as a normal response in the negative feedback mechanism. As the FSH level in Wi-Fi and Sham Wi-Fi groups was lower than in the Control group, we speculate that the serum level of FSH may have been affected by the Wi-Fi exposure rather than a product of a negative feedback mechanism. A more significant decrease was observed in the serum level of LH in the Wi-Fi and Sham Wi-Fi groups.

The reduction of both gonadotropin hormones suggested that Wi-Fi exposure has affected the pituitary gland. A similar finding was reported by Kaur & Khera, which reported the decrease of FSH and LH secretion following RFR exposure of a cell phone toward male albino rats (Khaur and Khera, 2018). Aside from FSH and LH, the other pituitary hormone known as adrenocorticotropic hormone (ACTH) was also reported to be reduced (Eskander et al., 2012). As the average plasma level will remain constant from the start of puberty (Nassar et al., 2020), Wi-Fi exposure seems to “reprogram” the pituitary hormone and cause it to be secreted at a low level. Because this involves a region of the brain, the findings of this study and other previously confirmed evidence should raise worrisome concerns about the implications of Wi-Fi or cell phone use, particularly among children.

Interestingly, the serum testosterone level was not affected by Wi-Fi exposure. Despite the low serum LH level, the Leydig cells are still responsive and secrete testosterone. According to Santi et al. (2020) relatively low levels of LH may be adequate to maintain intra-testicular testosterone with the presence of FSH. This observation seems to confirm our LH and testosterone findings. The testosterone levels in the Wi-Fi group were similar to the control groups may have contributed to the testis enlargement during puberty, as noted in the preceding paragraph.

### Effect of Edible Bird’s Nest Supplementation in Wi-Fi Exposed Rats

Supplementation of 250 mg/kg EBN was given to evaluate its attenuation effect on the Wi-Fi exposure. However, the testis coefficient in the Control EBN group showed a significant decrease compared to the Control group. The same result was recorded in the Wi-Fi + EBN group, which showed a significant reduction in testis and epididymal coefficients. These data indicate that supplementation of EBN 250 mg/kg to the pre-pubertal young has decreased testis and epididymal coefficients. This result contrasts with the supplementation of the EBN 250 mg/kg in the adult rats group, which did not cause any decrease in testis and epididymal coefficients (Jaffar et al., 2021).

The decrease in testis and epididymal organ coefficients following EBN supplementation to the young rats was likely due to estradiol (E2) hormone activity. This hormone was previously characterized to present in the EBN extract used in this study (Jaffar et al., 2021). Several previous studies have reported an association between the administration of the hormone E2 on the development of the reproductive system of male rats. These include significant testicular weight loss (Kula et al., 2001; Brouard et al., 2016), epididymis, and seminal vesicles (Goyal et al., 2003) when mice received E2 or other forms of estrogen. The decrease in organ weight is closely associated with a decrease in the percentage of seminiferous tubules with lumen (Brouard et al., 2016). Furthermore, the percentage of apoptosis in the Sertoli cell and spermatogonia was found to increase. This was accompanied by a decrease in the number of spermatocytes (Walczak-Jȩdrzejowska et al., 2013).

However, the changes in the testis microenvironment showed otherwise. The spermatogonia mitosis status in the Control EBN group showed no significant differences compared to the Control group. Yet, EBN supplementation in the Wi-Fi + EBN group showed an increase in the mitotic status of spermatogonia. These findings suggest that EBN can preserve and improve spermatogonia’s proliferation, which is affected by Wi-Fi exposure. Several previous studies have shown that EBN can promote cell proliferation *in vitro*. EBN capability includes the proliferation of rabbit corneal keratocyte cell culture (Abidin et al., 2011) and human adipose-derived stem cells (hADSCs) (Roh et al., 2012). In *in vivo* studies, EBN supplementation has been reported to accelerate the proliferation and activation of B cell antibodies (Zhao et al., 2016) and stimulate the proliferation of uterine structures (Albishtue et al., 2018).

The study done by Roh et al. (2012) has reported that activation of cell proliferation by EBN occurs through mitogen-activated protein kinase (MAPK). MAPK is also among the activated pathways when c-Kit autophosphorylation occurs following SCF binding (Cardoso et al., 2014). Therefore, it can be assumed that the increase in spermatogonia mitosis status in this Wi-Fi + EBN group is due to the improvement in the expression of c-Kit and SCF proteins. Furthermore, our data suggest that it further enhances MAPK activation contributes to increased spermatogonia proliferation. However, the mechanisms involved in the activation of these downstream road sites are beyond the scope of the current study.

Evaluation of the c-Kit-SCF regulatory factor showed an insignificant increase in c-Kit and SCF mRNA expression of the Control EBN group compared to the Control group. The small changes in c-Kit and SCF mRNA expression did not provide any significant differences in the expression of c-Kit and SCF proteins in the Control EBN group. EBN supplementation in the Wi-Fi + EBN group also showed an increase in c-Kit mRNA expression compared to the Wi-Fi group. The SCF mRNA expression also showed an improvement compared to the Wi-Fi group. Consistent with these mRNA changes, the expression of both c-Kit and SCF proteins in the Wi-Fi + EBN group increased compared to the Wi-Fi group. Nevertheless, changes in the expression patterns of c-Kit and SCF proteins in the Wi-Fi + EBN group were insignificant compared to the Wi-Fi group. To some extent, supplementation of EBN showed that it could prevent the deterioration of the mRNA expression of c-Kit and SCF in the testis receiving Wi-Fi exposure.

The second spermatogonia proliferation regulatory factor showed a significant decrease in gonadotropin hormone levels following EBN supplementation. A significant reduction was recorded in LH hormonal levels. However, EBN supplementation did not cause a significant reduction in FSH and T serum hormonal levels. From these findings, EBN supplementation had caused a decrease in gonadotropin hormone levels, especially in LH hormonal levels. The reduction in LH hormone following EBN administration in rats that had not reached puberty was also reported to be closely related to E2 hormone activity (Gill-Sharma et al., 2001). Thus, the considerable decrease in LH was the second indication of E2 activity in EBN after significantly decreasing the testis and epididymis coefficient. As a result, despite EBN potential as a male fertility treatment, EBN consumers must be cautious of the presence of this specific hormone, which may produce adverse effects, especially in pre-pubertal children.

However, EBN supplementation to the Wi-Fi + EBN group had increased FSH hormonal levels. The mechanism by which EBN increases the FSH in this group is yet to be understood. The probable factors of the increase in serum FSH level may be due to the presence of FSH in the EBN itself (Ma and Liu, 2012b) or the EBN has stimulated FSH production after spermatogonia proliferation was found to be decreased due to Wi-Fi exposure. However, the pathway involved are unclear and need to be further evaluated in future studies. Somehow, the increase in the spermatogonia mitotic status observed in the Wi-Fi + EBN group was most likely due to the elevated FSH levels in this serum together with restoration of gene expression and c-Kit and SCF.

## Conclusion

The long-term exposure of developing rat pups to Wi-Fi throughout the pre-pubertal age to adulthood has detrimental effects on their reproductive development. Although the Wi-Fi exposure did not significantly reduce the c-Kit-SCF proliferative regulatory system, it had decreased the spermatogonia mitosis activity. It is essential to note that the decrease in the spermatogonia mitosis status may be partly due to the effect of Wi-Fi on the hypothalamus-pituitary-gonadal (HPG) axis which caused the reduction in the gonadotropin hormone. This result was accomplished by using a 64 bps ping signal, significantly less than the amount of data traffic we encounter in our daily lives. Email, for example, requires at least 0.5 Mbps, video requires 1.5 Mbps, and HD video or two-way video games demand 4.0 Mbps (Liu et al., 2018). A more severe effect could be expected because of the higher data transfer rate in our daily lives. However, until more research is done, it will remain a hypothesis. These findings may reflect the progression of infertility in children upon reaching reproductive age due to Wi-Fi exposure. Therefore, the use of Wi-Fi among children should be minimized due to the risk associated with prolonged radiation exposure.

Attenuation of long-term Wi-Fi exposure in young children with EBN had demonstrated a promising finding where it increases the spermatogonia proliferation despite the Wi-Fi exposure given. However, EBN supplementation at a dose of 250 mg/kg per day on young rats from pre-pubertal age until adulthood was found to have side effects. The side effect includes decreased testis and epididymis coefficient and decreased gonadotropin, LH, and FSH. It is most likely related to the hormone E2, which is also present in the EBN. Therefore, despite the high potential of EBN as a treatment for male infertility, the results presented in this study indicate that there is a need to monitor and verify various health claims made against EBN. This cautionary advice is not only for user’s protection but also for ensuring the EBN can be developed safely for human consumption without any unwanted side effects.

## Data Availability Statement

The original contributions presented in the study are included in the article/supplementary material, further inquiries can be directed to the corresponding author/s.

## Ethics Statement

The animal study was reviewed and approved by the UKM Animal Ethical Committee (FISIO/PP/2018/SITI FATIMAH/28-MAR./908-MAR.-2018-DEC.-2020).

## Author Contributions

FJ, SI, and KO designed the study. FJ and AZ conducted the data acquisition, interpreted, and analyzed the data. FJ drafted. SI, KO, and CH revised the manuscript and supervised the whole work. All authors read and approved the final manuscript.

## Conflict of Interest

The authors declare that the research was conducted in the absence of any commercial or financial relationships that could be construed as a potential conflict of interest.

## Publisher’s Note

All claims expressed in this article are solely those of the authors and do not necessarily represent those of their affiliated organizations, or those of the publisher, the editors and the reviewers. Any product that may be evaluated in this article, or claim that may be made by its manufacturer, is not guaranteed or endorsed by the publisher.
